# Letter to the Editor regarding ‘Evaluating nurse preferences for a novel on-body delivery system vs. manual syringes for large-volume subcutaneous drug administration: a survey study’

**DOI:** 10.1080/10717544.2025.2537813

**Published:** 2025-08-04

**Authors:** Rohan Medhekar, Laura Clark, Santosh Gautam, Annelore Cortoos

**Affiliations:** Johnson & Johnson, Horsham, PA, USA

We read with great interest the article by Desai et al. (2025), which explored nurses’ preferences for administering large-volume subcutaneous drugs using a hypothetical on-body delivery system (OBDS) versus a manual syringe. We acknowledge the authors’ effort to give nurses a voice in the selection of preferred drug delivery methods for oncology drugs. However, significant issues concerning the validity, interpretation, and applicability of this research warrant further examination.

First, the participants were asked to state their preferences for using a hypothetical OBDS for large-volume subcutaneous drug administration after watching a 30-second video and a four-minute presentation, compared to using manual injections with which they have extensive hands-on experience. This approach raises concerns about hypothetical bias, as participants’ responses in hypothetical scenarios can differ from their real-world behaviors (Buckell et al. 2020). Due to the lack of real-world experience with OBDS and the brevity of the video/presentation on OBDS, the potential complexities of OBDS may not have been fully captured for a fair comparison, leading to an overestimation of OBDS’s benefits and a possible misrepresentation of the nurses’ true preferences. These limitations should be acknowledged.

Second, there are methodological concerns with participant selection, and the reliability and validity of the survey questionnaire. The sampling process is unclear, and selection bias may have occurred. The number of nurses in the 1.5 million respondent database was not reported. The survey targeted all U.S. nurses in the database, yet only a small sample of 45 nurses responded, limiting generalizability. The reasons for non-responses and the number of incomplete responses were not reported. Additionally, the rationale and significance of selecting the initial 13 nurses with experience administering daratumumab/hyaluronidase was not explained. Further, no information was provided on the questionnaire validation process, which is crucial to ensure precise measurement and to avoid ambiguities in the questionnaire that might impact the survey results (Cobern and Adams 2020).

Third, in the detailed, drug-specific scenarios presented in Table 2 of the article, key parameters such as skin preparation (e.g. shaving for OBDS adhesion), suitability for patients with skin conditions, and challenges related to skin folds were excluded. The study also failed to address issues like OBDS sterility, storage requirements, and biohazard waste management.

Lastly, the study introduced ambiguity by not clarifying whether the ‘Drug X’ administered with OBDS refers to the oncology drugs – daratumumab, rituximab, and the pertuzumab/trastuzumab combination – or a completely different medication. These oncology drugs have transformed the treatment paradigm for millions of cancer patients across the globe, e.g. daratumumab has demonstrated deep and durable responses, extended progression free survival as well as overall survival in multiple myeloma patients across all ages, fitness and risk profiles while maintaining a tolerable safety profile (Facon et al. 2024; Sonneveld et al. 2024). If the study participants erroneously assumed these oncology drugs were delivered using the hypothetical OBDS, it could have skewed their preferences.

In conclusion, Desai et al. do not provide a balanced view of their findings, as they overlook critical limitations of their study and disproportionately highlight the advantages of the hypothetical OBDS compared to the well-established large-volume subcutaneous drug administration using manual syringes.

## Data Availability

Data sharing is not applicable to this article as no datasets were generated or analyzed for this publication.
